# Hyaluronic acid-modified didecyldimethylammonium bromide/ d-a-tocopheryl polyethylene glycol succinate mixed micelles for delivery of baohuoside I against non-small cell lung cancer: *in vitro* and *in vivo* evaluation

**DOI:** 10.1080/10717544.2016.1228713

**Published:** 2017-02-03

**Authors:** Hongmei Yan, Jie Song, Xiaobin Jia, Zhenhai Zhang

**Affiliations:** 1 College of Pharmacy, Nanjing University of Chinese Medicine, Nanjing, PR China and; 2 Key Laboratory of New Drug Delivery System of Chinese Materia Medica, Third School of Clinical Medical of Nanjing University of Chinese Medicine, Nanjing, PR China

**Keywords:** Baohuoside I, mixed micelles, hyaluronic acid, antitumor, targeting

## Abstract

Baohuoside I is an effective but a poorly soluble antitumor drug. In this study, we prepared baohuoside I-loaded mixed micelles with didecyldimethylammonium bromide (DDAB) and d-a-tocopheryl polyethylene glycol succinate (TPGS) (DTBM) and active targeting mixed micelles (HDTBM) with hyaluronic acid (HA) as the targeting ligand on the surface of the mixed micelles. We performed a systematic comparative evaluation of the antiproliferative effect, cellular uptake, antitumor efficacy, and *in vivo* tumor targeting of these micelles using A549 cells. HDTBM showed improved cellular uptake and had a greater hypersensitizing effect on A549 cell lines than baohuoside I; half-maximal inhibitory concentration (IC_50_) was 8.86 versus 20.42 μg/mL, respectively. Results of the antitumor efficacy study and the imaging study for *in vivo* targeting showed that the mixed-micelle formulation had higher antitumor efficacy and achieved effective and targeted drug delivery. Therefore, our results indicate that HA/baohuoside I-M may be used as a potential antitumor formulation.

## Introduction

Baohuoside I (also known as icariside II) is the main active flavonoid component of Herba epimedii, which has been traditionally used in China as a tonic, an aphrodisiac and an antirheumatic drug for many years (Qian et al., 2012; Yu et al., 2013; Zhai et al., 2013; Xiao et al., 2014). Baohuoside I induces apoptosis in human non-small cell lung cancer cells via reactive oxygen species-mediated mitochondrial pathway and inhibits the growth of U266 multiple myeloma and pre-osteoclastic RAW264.7 cells (Choi et al., 2008; Kim et al., 2011; Jin et al., 2012a; Song et al., 2012; Liu et al., 2014).

Baohuoside I is poorly soluble in water. Moreover, baohuoside I has low absorptive permeability and a high rate of efflux via apical efflux transporters such as multidrug resistance-associated protein 1 and 2 (MRP 1, MRP 2) and P-glycoprotein (P-gp); therefore, the use of baohuoside I in the management of human ailments has been restricted (Chen et al., 2008). While the membrane permeability of baohuoside I is slightly better than that of the other flavonoids in Herba epimedii, the rate of efflux (*P*_BA_/*P*_AB_) of baohuoside I reached 9.84 in a previous study using Caco-2 cells. Thus, poor aqueous solubility, low membrane permeability, and a severe efflux phenomenon limit the therapeutic use of baohuoside I in humans (Jeong et al., 2005; Jin et al., 2012b).

Therefore, improvement in the aqueous solubility and membrane permeability and reduction of the efflux phenomenon of baohuoside I are both important for determining the future applications of baohuoside I. In previous studies, various drug delivery systems, including phospholipids complexes and nanoparticles, have been developed to overcome the aforementioned limitations of baohuoside I (Jin et al., 2012a,b). Among the several micellar formulations evaluated as carriers of anticancer drugs, polymeric micelles are the carriers of choice of baohuoside I. Micellar systems have many advantages such as increasing the drug solubility, circumventing the uptake by the reticuloendothelial system (RES), improving circulation time, and passive tumor targeting by the enhanced permeability and retention (EPR) effect (Fang et al., 2011; Guerry et al., 2014; Sarika et al., 2015).

Mixed micelles have synergistic properties such as increased drug stability and drug loading efficiency compared to that of the individual components (Potluri et al., 2006; Pooja et al., 2014; Zhang et al., 2014). To the best of our knowledge, no studies have shown the influence of mixed micelles for the delivery of baohuoside I. Therefore, we prepared mixed micelles of baohuoside I consisting of didecyldimethylammonium bromide (DDAB) and d-a-tocopheryl polyethylene glycol succinate (TPGS) and modified by hyaluronic acid (HA) to increase the aqueous solubility and membrane permeability and limit the efflux of baohuoside I.

HA has been used a vector for the delivery of anticancer drugs and has become the hotspot of tumor targeted drug delivery systems (Kumar et al., 2015; Qi et al., 2015). The HA receptor CD44 and receptor for hyaluronan-mediated motility (RHAMM) are highly expressed on the surface of tumor cells. HA has the ability to recognize the specific receptors, which are overexpressed on the surface of tumor cells, and it can facilitate targeted delivery of the anticancer drug to the tumor cells (Surace et al., 2009). The pKa of carboxyl of D-aureus in HA was 3–4, and most of the ions were ionized in physiological conditions (pH 7.4); therefore, HA was almost in the form of multi anions.

Vitamin E TPGS or simply TPGS is a water-soluble derivative of natural vitamin E, which is formed by esterification of vitamin E succinate with polyethylene glycol (PEG). In the recent years, TPGS has been extensively used for developing various drug delivery systems and extending the half-life of drugs in the plasma and increasing the cellular uptake of drugs (Rajebahadur et al., 2006; Wang et al., 2015). Therefore, TPGS can be used as an ideal molecular biomaterial for developing various drug delivery systems, including prodrugs, micelles, liposomes, and nanoparticles, which would enable sustained, controlled, and targeted drug delivery. Moreover, TPGS has been used as an excipient for overcoming multidrug resistance (MDR) and as an inhibitor of P-gp (Zhang et al., 2012).

DDAB is a double chain cationic surface-active agent. It has both hydrophilic and hydrophobic groups. Supramolecular self-assembly structures such as micelles, microemulsions, and vesicles in solution can be formed because of hydrophobic interactions in DDAB. The study by Kusumoto indicates that DDAB is a potent inducer of cell death in a wide range of tumor cell lines; their results show that leukemia cells (HL-60 and U937) and neuroblastoma cells (Neuro2a) were more sensitive to DDAB than carcinoma cells such as HepG2 and Caco-2 cells (Kusumoto & Ishikawa, 2010). Because of the electrostatic effect, positively charged particles are easily deposited in the lung, and the target of the lung is high. Unlike HA, DDAB has a catioinic structure, which plays an active role in the surface activity. HA can be attracted by DDAB via positive and negative charges. The DDAB cationic carrier modified by HA can shield the excess cations and reduce the cytotoxicity of the micelles. Moreover, the compound formulated can give a targeting ability to the micelles.

To increase the solubility and membrane permeability and enhance the anti-cancer effect of baohuoside I by limiting its efflux, we prepared baohuoside I-loaded mixed micelles consisting of DDAB and TPGS (DTBM) and HA-modified baohuoside I-loaded DDAB/TPGS mixed micelles (HDTBM) by using a thin-film hydration method and compared them with each other. We selected the human lung adenocarcinoma cell line A549 to investigate the *in vitro* and *in vivo* anticancer effects of mixed micelles. In addition, we examined the physicochemical properties and *in vivo* targeting behavior of the baohuoside I-loaded micelles in this study.

## Materials and methods

### Materials

Baohuoside I was obtained from the Nanjing Zelang Medical Technology Co. Ltd (Nanjing, PR China) and purity was > 98%. TPGS, DDAB, and HA were purchased from Aladdin Industrial Co. Ltd (Liguria, Italy). Plastic cell culture dishes and plates were purchased from Corning Incorporated (Corning, NY). Coumarin-6 and DAPI were purchased from Sigma-Aldrich (Shanghai, PR China). DiR was purchased from Ganhua Trade Co. Ltd. (Shanghai, PR China). All reagents were of analytical grade except methanol, which was of chromatographic grade.

### HPLC analysis

The concentration of baohuoside I in the dissolution medium was determined by high-pressure liquid chromatography (HPLC, Agilent 1260) equipped with a Diamonsil™ RP-C18 column (250 × 4.6 mm, 5 μm). The mobile phase of methanol and water (75:25, v:v) was used at a flow rate of 1.0 mL·min^−1^. The UV detector was set at 270 nm to analyze the column effluent and the column temperature was 30 °C. The entire solution was filtered through a 0.45 μm membrane filter (Merck Millipore Corp., Billerica, MA) and degassed prior to use. The injection volume was 10 μL. The recovery rates for baohuoside I were in the range of 99–101% and the RSD were less than 2%. Intra-day and inter-day precisions for baohuoside I were below 2%.

### Preparation of baohuoside I -loaded mixed micelles

Baohuoside I -loaded mixed micelles were prepared by a thin-film hydration method (Tong et al., 2012). The compositions of different micelles are shown in Table 1. Briefly, baohuoside I and micelles materials (1:5, w/w) were co-dissolved in ethanol in a round-bottom flask. The solvent was subsequently evaporated by rotary evaporation to obtain a thin film. The film was then kept in a vacuum overnight at room temperature to remove the residual ethanol. The dried film was hydrated with 0.9% NaCl solution and a clear micelle solution was formed. At last, the fully swollen HA was added into the micelle solution by stirring till mixing. The solution was then filtrated through 0.22 μm membrane filter to remove the non-incorporated baohuoside I, followed by lyophilization (Liu et al., 2008).

**Table 1.** Physicochemical characterizations of baohuoside I-loaded micelles (*n *= 3).

Formulation	Composition (molar ratio)	Size (nm)	Zeta potential (mV)	Encapsulation (%)	Drug loading (%)
DTBM	DDAB/TPGS (1:4)	22.63 ± 1.09	16.74 ± 2.33	88.36 ± 6.03	16.14 ± 0.44
HDTBM	HA/DDAB/TPGS (1:5:20)	31.42 ± 1.15	−1.25 ± 0.35	86.78 ± 4.88	15.87 ± 0.41

### Baohuoside I loading efficiency and encapsulation ratio

The content of the baohuoside I was determined by HPLC assay after diluting the micelles with ethanol. All samples were analyzed in triplicate. The encapsulation efficiency and the drug loading efficiency were calculated using the following equations:

(1) $$\mathrm{Encapsulation}\;\mathrm{efficiency}\left(\%\right)\;\;=\;\frac{\text{weight of baohuoside I in micelles }}{\text{weight of baohuoside I fed initially}}\;\times \;100\%$$

(2) $$\text{Drug loading efficiency (}\%)\;\;=\;\;\frac{\text{weight of baohuoside I in micelles}}{\text{total weight of micelles}}\;\times \;100\%$$

### Characterization of baohuoside I -loaded mixed micelle

The size (hydrodynamic diameter) and zeta-potential of all micelles were measured using a Zetasizer Nano ZS (Malvern Zetasizer 3000; Malvern Instruments Ltd, Malvern, UK). The particle size and zeta-potential measurements were carried out in triplicate. The morphological evaluation was performed using transmission electron microscopy (TEM; H7650; HITACHI, Tokyo, Japan). The micellar solution was diluted in the concentration of 100 μg/mL and stirred for 24 h. A few drops of sample was taken to film the copper net formation of droplets and dried in the natural air. It was negative stained with phosphomolybdic acid and observed under the transmission electron microscope. Pyrene was used as a fluorescence probe to determine the CMC values of micelle. Typically, the micelle solutions at various concentrations were added to the containers with pyrene. The concentration of pyrene in solution was 6 × 10^−7^ M. Thereafter, these samples were equilibrated at room temperature overnight. The intensity ratio of I338/I333 was recorded as a function of polymer concentration.

### *In vitro* baohuoside I release

The *in vitro* release behavior of baohuoside I from baohuoside I mixed micelles was monitored in a phosphate buffered saline (PBS) (PH7.4) medium containing 0.5% Tween 80 to obtain pseudo sink conditions. Briefly, aliquots of baohuoside I mixed micelles were introduced into a dialysis bag (MWCO = 3500 Da, Green bird Inc, Shanghai, PR China), the sealed end of which was immersed fully into 50 mL of a release medium at 37 °C; this medium was stirred at 100 rpm for 24 h. At fixed time intervals, 0.5 mL aliquots were withdrawn and replaced with an equal volume of fresh medium. Baohuoside I releases from the stock solution was conducted under the same conditions as the controls. The concentration of baohuoside I in the samples was determined by HPLC as described above, with correction for the volume replacement.

### Cell lines and cell culture

The human lung adenocarcinoma cell line A549 was obtained from Nanjing KeyGEN Biotech. Co. Ltd (Nanjing, PR China), which was cultured with RPMI 1640 contained 10% FBS, 100 IU/mL penicillin and 100 μg/mL streptomycin sulfates at 37 °C in 5% CO_2_.

### Animals and tumor implantation

Male BALB/c nude mice (22 ± 2 g) were obtained from Changzhou Cavens Lab Animal Co. Ltd (Changzhou, PR China). All animal experiments were performed in accordance with protocols evaluated and approved by Jiangsu Provincial Academy of Chinese Medicine’s Experimental Animal Center. Pathogen free BALB/c mice were housed in separate cages with normal access to food and water and kept on a 12 h light-dark cycle. To generate tumors, the flanks of six-week-old male BALB/c mice were shaved and 200 μL of single cell suspension containing 1 × 10^6^ A549 cells in serum-free DMEM was injected subcutaneously under anesthesia.

### *In vitro* cytotoxicity assay

The cytotoxicity of baohuoside I, Paclitaxel, DTBM and HDTBM against A549 cells was measured using the MTT assay. The cells were seeded into a 96-well plate 100 μL of single cell suspension containing 1 × 10^5^ A549 cells. After incubation for 24 h, the cells were treated with various baohuoside I formulations at a range of concentrations for 24, 48 and 72 h. Then 10 μL MTT (5 mg/mL) was added to each well. After incubation for 4 h, the medium was removed and replaced with 100 μL DMSO solution to dissolve. The absorbance of each well was measured by a SpectraMax 190 microplate reader (Molecular Devices, Sunnyvale, CA) at the wavelengths of 570 and 630 nm. Each experiment was repeated three times.

### *In vitro* cellular uptake: quantitative study and qualitative study

#### Quantitative study

The A549 cells were seeded into a six-well culture plate. After incubation for 24 h, the original culture medium was removed and the cells were treated with baohuoside I formulations at a concentration of 25 μM for 1, 2 and 4 h incubation to evaluate the effects of the relationship between uptake and uptake time. After the incubation, the medium was fast discarded, and the cold PBS was ingested and the cells were washed three times. Then 1 mL of distilled water was added and cells were scraped. The suspended fluid of cells were collected and placed in an ice bath under ultrasonic cell disruption, centrifuged, and the supernatant was in another clean centrifuge tube, dried with nitrogen, dissolved in methanol, with 0.45 μm millipore filtration, constant volume to 1 mL, and used HPLC determination to measure the content of baohuoside I.

#### Qualitative study

*In vitro* qualitative study of cellular uptake of the micellar formulations was assessed by the fluorescence microscope. After reaching confluence, cells were detached, counted and seeded in a 24-well plate overnight. Then, the medium was replaced by coumarin-6 loaded micelles suspension at concentration of 0.2 mg/mL and incubated for 2 h. The cells were washed twice with pre-warmed PBS and fixed with 95% ethanol for 20 min. After that, the cells were washed twice with PBS and then the nuclei were counterstained by DAPI for 30 min. The cells were washed again twice by PBS and immersed in PBS for microscopic imaging.

### *In vivo* antitumor activity evaluation

For *in vivo* implantation, nude mice were subcutaneously injected in the right flank with 0.2 mL of cell suspension containing 1.7 × 10^6^ A549 cells. The *in vivo* antitumor studies were started when the tumor volumes reached about 60 mm^3^ (designated as day 0). Mice were randomly divided into five groups (*n *= 6): group 1 for saline solution, group 2 for Paclitaxel injection (10 mg/kg), group 3 for baohuoside I (10 mg/kg), group 4 for DTBM (10 mg/kg) and group 5 for HDTBM (10 mg/kg), respectively. Mice were administered according to their group protocol, through the tail vein for five times every two days. Tumor volume and mouse weight were monitored daily. Tumor volume was calculated by the equation *V* = (*a*×*b*^2^)/2, where *a* represents the longest diameter and *b* represents the shortest diameter perpendicular to length. At the end of the experiment, the animals were sacrificed and the tumor, thymus and spleen masses harvested, and weighed. HE staining was performed to evaluate the safety of the micelles. The mice were sacrificed by cervical dislocation, and the liver and kidney tissue were taken and fixed in fresh 10% formaldehyde-mixing fixative for 24 h, rehydrated, and embedded into paraffin. Serial 5 mm sections were cryosectioned and then placed on superfrost/plus microscope slides. The sections were stained with HE according to the manufacturer’s protocol, then observed and photographed with a digital camera.

### *In vivo* imaging

DiR-M (DiR-loaded mixed micelles composed of DDAB and TPGS) and HA/DiR-M (DiR-loaded mixed micelles composed of DDAB and TPGS and modified by HA) were prepared by a thin-film hydration method according the prescription of baohuoside I-loaded mixed micelles. When tumors reached an acceptable size, DiR-loaded mixed micelles were injected into the tail vein of the tumor-bearing mice at a dose of 5.0 mg/kg to investigate the *in vivo* distribution. The mice were anesthetized and imaged at the predetermined time (0.5, 2, 4, 8, 12 and 24 h) after intravenous injection using the Maestro *in vivo* imaging system (NightOWL II LB983, Berthold, Germany).

### Statistical analysis

Data of the above experiments were expressed as mean ± standard deviation (SD). Statistical analysis was performed by Kruskal–Wallis test. *p *< 0.05 was considered as statistically significant.

## Results

### Formulation and characterization of mixed micelles

**Figure 1.**
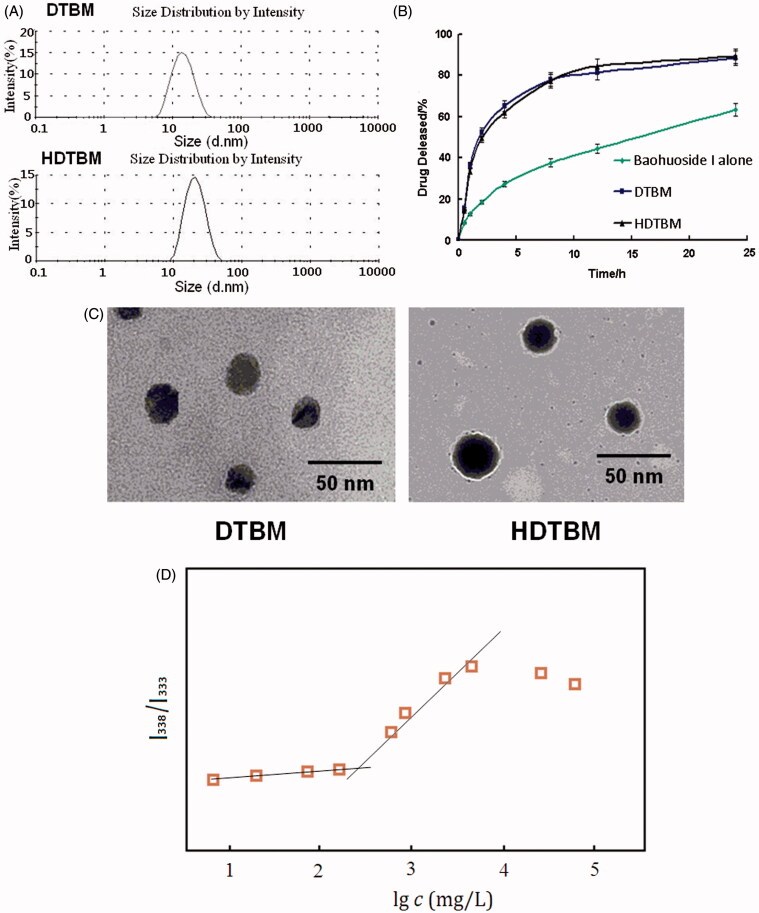
Characteristics of the baohuoside I-loaded micelles. Size distribution of baohuoside I-loaded micelles (A). Baohuoside I release profiles from the micelles *in vitro* (B).Transmission electron microscope (TEM) image of baohuoside I-loaded micelles in 50 nm scale (C). Quotient of vibrational band intensities (I_338_/I_333_) from excitation spectra of pyrene as a function of lg *c* of mixed micelle in distilled water (D). Data are presented as the mean ± SD (*n *=3).

We prepared baohuoside I-loaded micelles by using the film formation method. The mean diameter of baohuoside I-loaded micelles determined using Zetasizer Nano ZS and TEM was ∼22–30 nm (Table 1, Figure 1A and 1C). The absolute zeta-potential values of the DTBM were positive. HDTBM was negatively charged with an absolute zeta-potential of 1.25 mV. Encapsulation efficiencies of these micelles were above 85%. We observed no significant differences in the size, morphology and encapsulation efficiencies of these two baohuoside I-loaded mixed micelles.

The accumulated percentage release of baohuoside I from the active pharmaceutical ingredient baohuoside I, DTBM and HDTBM in PBS (pH 7.4) containing 0.1% (w/v) Tween 80 is shown in Figure 1(B). Baohuoside I showed an initial burst release in 4 h. DTBM and HDTBM showed controlled release for more than 1 d without any burst release. Moreover, both mixed micelles showed a similar release behavior. After 24 h of dialysis in PBS (pH 7.4), the percentage of baohuoside I released from the mixed micelles was 82.43 and 74.18% for DTBM, and HDTBM, respectively. Compared to baohuoside I, the mixed micelles (DTBM and HDTBM) showed a significant (*p *< 0.05) sustained release in the *in vitro* drug release.

To establish the CMC of resulting micelles, pyrene was used as fluorescence probe, and the intensity ratios of them at I_338_/I_333_ were measured against the logarithm of the polymer concentration. As shown in Figure 1(D), the point of slope changed is the critical micelle concentration of mixed micelle. Its CMC is 231.25 mg/L which is much lower than those commonly used low molecular surface-active agents (e.g. The CMC of Poloxamer is 1.0 × 10^3^ ∼2.4 × 10^4^ mg/L). Generally speaking, lower CMC can make the concentration of aggregates formed be higher at the same polymer concentration. It will provide more hydrophobic reservoir for drugs which are poorly soluble in water and better dilution stability.

### *In vitro* evaluation of baohuoside I micelle formulations

We examined the *in vitro* cytotoxicity of baohuoside I formulated in baohuoside I, DTBM, and HDTBM and compared it with that of paclitaxel at an equivalent concentration on A549 cells after incubation at 37 °C for 24, 48 and 72 h (Table 2). The mixed micelle formulations (DTBM and HDTBM) had lower IC_50_ values than baohuoside I, which indicated that compared to the free drug, the mixed micelle formulation could significantly increase the cytotoxicity of baohuoside I against A549 cells *in vitro*. Moreover, HDTBM was more cytotoxic than DTBM at equivalent drug concentrations. In addition, mixed micelles without the drug showed antiproliferative effects when the concentration of micellar materials was higher than 50 ug/mL.

**Table 2.** IC_50_ values (μg/mL) of free baohuoside I or baohuoside I-loaded micelles against A549 cells (*n *= 6).

	IC_50_ (μg/mL)				
Incubation time/h	Paclitaxel	Baohuoside I	DTBM	HDTBM	Micelles without baohuoside I
24	6.28 ± 0.21	20.42 ± 1.37	13.71 ± 0.58	8.86 ± 0.43	78.32 ± 3.27
48	4.46 ± 0.17	16.35 ± 1.41	9.54 ± 0.45	5.39 ± 0.36	69.45 ± 3.04
72	4.01 ± 0.09	14.63 ± 1.18	6.15 ± 0.29	3.24 ± 0.20	58.17 ± 0.25

**Figure 2.**
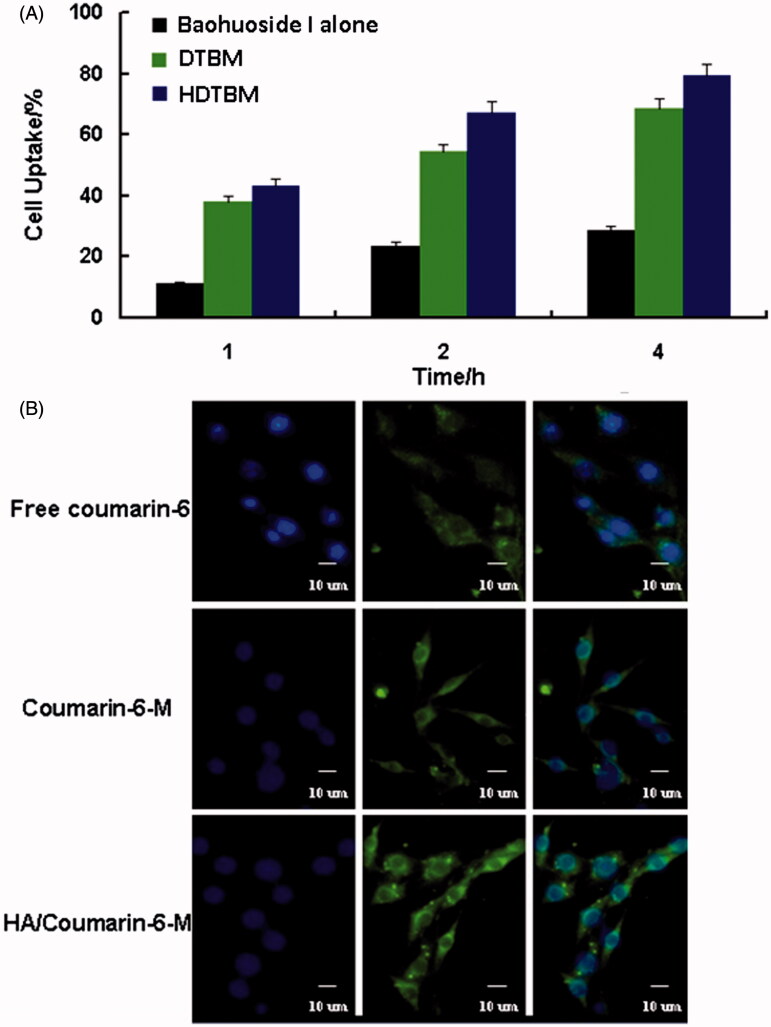
Cellular uptake efficiency of the baohuoside I and baohuoside I-loaded micelles by A549 cells after 1, 2 and 4 h incubation (A). Fluorescence microscope of A549 cells after 2 h incubation with the free fluorescent coumarin-6 and the coumarin-6-loaded mixed micelles (B).

The quantitative uptake by A549 cells after incubation with coumarin-6 loaded mixed micelles for 1, 2 and 4 h is shown in Figure 2(A). We observed a trend of increased uptake of mixed micelles by the cells with an increase in the incubation time. The cellular uptake of the two mixed micelle formulations was significantly higher than that of the free drug. In addition, the cellular uptake of HDTBM was higher than that of DTBM during the same incubation time. The qualitative uptake by A549 cells after incubation with the coumarin-6 loaded mixed micelles for 2 h is shown in Figure 2(B). For enabling comparison of the intensity of fluorescence among the cells treated with the three types of baohuoside I formulations, we acquired the images under the same imaging parameters such as sensitivity, gain, offset and laser power constant throughout the cell imaging process. Our results showed that the green fluorescence in the A549 cells, which corresponds to the coumarin-6-mixed micelles loaded by DDAB and TPGS (coumarin-6 M) and HA-modified coumarin-6-mixed micelles (HA/coumarin-6 M), was stronger than that of the free coumarin-6. More regions in the cytoplasm were stained in green, implying enhanced uptake of the HA-modified coumarin-6-mixed micelles (HA/coumarin-6 M).

### *In vivo* antitumor efficacy

**Figure 3.**
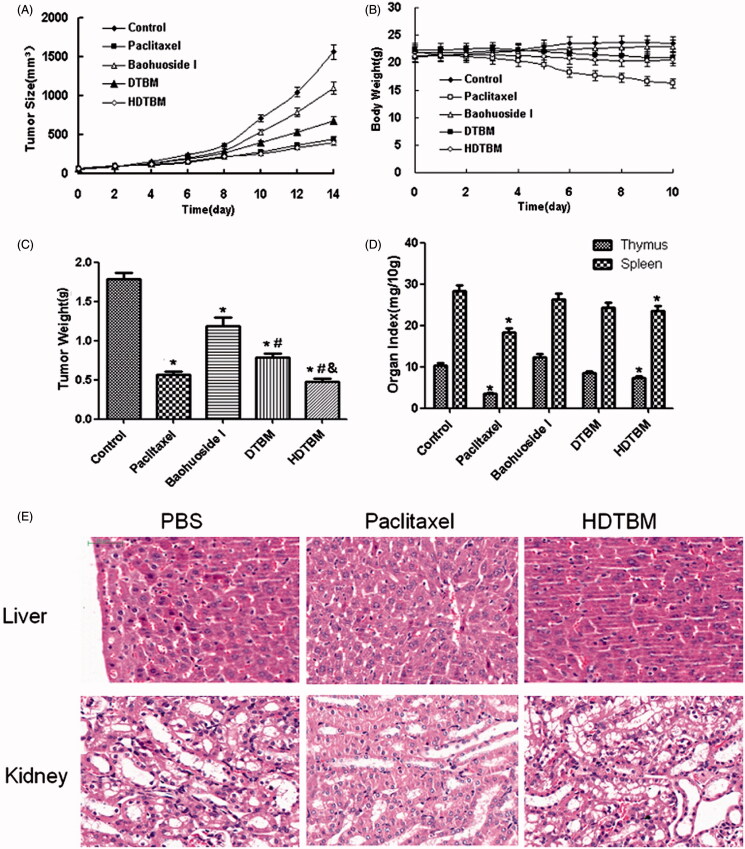
*In vivo* antitumor study of baohuoside I-loaded micelles in Balb/c nude mice implanted with A549 cells. Tumor volumes (A) and body weight (B) were monitored daily. Tumor weight (C) and organ index (D) were monitored at the end of the experiment. The results were presented as the mean ± SD (*n *= 6). *p <0.05, compared with control group. ^#^*p *< 0.05, compared with Baohuoside I group & *p *< 0.05, compared with DTBM group. H&E staining of liver and kidney sections excised from A549 tumor-bearing mice following 14 d treatment with HDTBM (E). Mice treated with paclitaxel and PBS were used as controls.

The micelles showed remarkable antitumor effects *in vitro*; therefore, we examined the antitumor effects of the micelles after systemic application. The tumor volume-time curve and weight analysis are shown in Figure 3(A) and 3(C). Our results showed that tumor growth in the control group was greater than that in the baohuoside I groups, which indicated that baohuoside I was effective anticancer drug. When compared to baohuoside I alone group, DTBM group and HDTBM group showed a moderate increase in tumor volume. This results confirmed that baohuoside I mixed micelles (DTBM and HDTBM) were efficient at inhibiting tumor growth to varying degrees. Because of the active targeting effect of HA, HDTBM produced stronger effects than DTBM, which could be concluded from the volume of tumors and weights.

The variations in the body weights of the mice were plotted against time as shown in Figure 3(B) and 3(D). The body weights of mice in the control group slightly increased, which could be related to the increasing tumor weight. The body weights of the mice treated with baohuoside I-loaded formulations decreased, which might be because of the impact of baohuoside I. The body weights of the mice in the paclitaxel group decreased to a greater extent than those in the baohuoside I-treated groups. Thus, baohuoside I-loaded formulations had lesser toxicity than paclitaxel. The results of organ index (percentage of organ weight to body weight of each mouse) in different groups are shown in Figure 3(D). Compared to the control group, the baohuoside-I group showed a decrease in the organ index of the thymus and spleen to varying degrees, which may be attributed to the inherent toxicity of baohuoside I, but the values remained within the tolerance range. Other organ indices did not show a significant difference between any groups. However, compared to the control group, the paclitaxel group showed a significant decrease in the organ index. H&E staining showed that HDTBM caused severe necrosis in the tumor tissue with little damage to the liver and kidney. In contrast, more significant liver and kidney damage were observed for mice treated with paclitaxel. These results confirm that HDTBM could actively target to CD44 positive tumor xenografts, kill cancer cells and spare the side effects of drug. Thus, our results showed that compared to paclitaxel, baohuoside I-loaded formulations had lesser toxic effects on normal tissues.

### Imaging to determine *in vivo* targeting of baohuoside I mixed micelles

**Figure 4.**
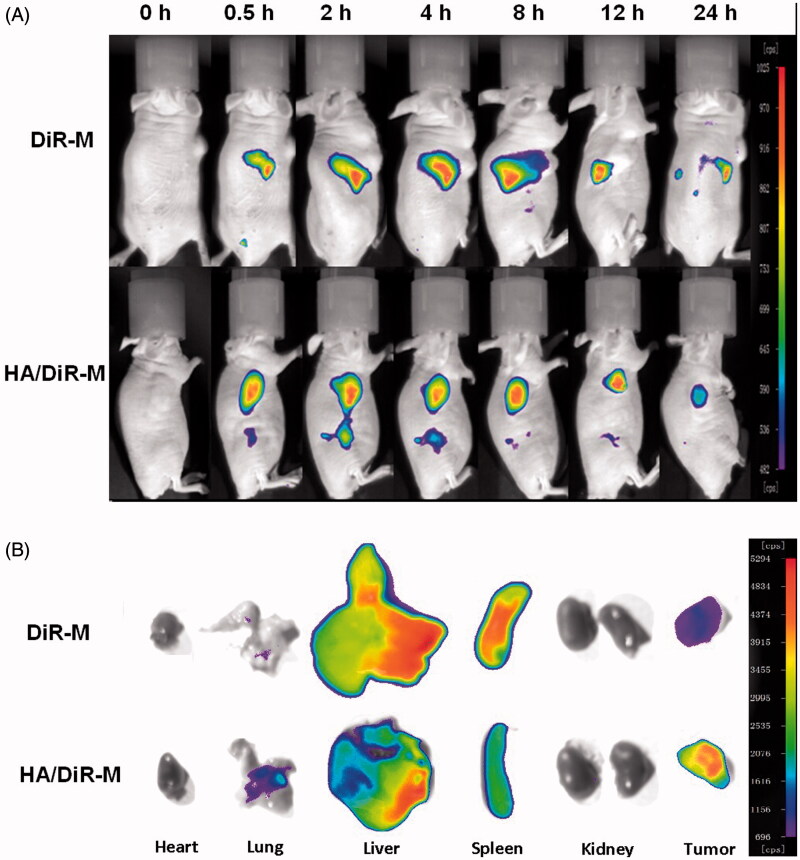
Fluorescence images of the mice bearing A549 cells on right sides at different time points after intravenous injection (A) and the distribution of DiR in various important organs (B).

To investigate whether HA/DDAB/TPGS mixed micelles can specifically target A549 cells *in vivo*, we established the tumor model by intravenously injecting mice with A549 cells in the right sides. Compared to mice treated with DiR-M, mice treated with HA/DiR-M showed a time-dependent increase in the fluorescence signals, and they were mainly distributed on the tumor (Figure 4A). Fluorescence signals were clearly observed at the tumor site and maintained for 24 h. Moreover, the distribution of DiR in various important organs was compared between DiR-M group and HA/DiR-M group (Figure 4B). Accumulation of DiR in liver and spleen was relatively lower for the DiR formulation, thus reducing the side effects from baohuoside I. While the accumulation of DiR in lung and tumor was higher for DiR formulation, which indicated that this formulation enabled delivery of chemotherapeutic drugs to solid tumors. Therefore, with the modification of HA and TPGS, the HA/DDAB/TPGS mixed micelles were efficiently delivered and retained by the lung tumors under *in vivo* conditions.

## Discussion

The anti-tumor effect of flavonoids has been extensively examined. Owing to their strong anticancer effects and low toxicity, flavonoids have a great potential of being developed as anticancer drugs (Ravishankar et al., 2013; Martinez-Perez et al., 2014; Orlikova et al., 2014). Baohuoside I is the main active flavonoid component of Herba epimedii and has pharmacological effects. Although baohuoside I has anti-tumor characteristics, it may be pumped out by the intestinal efflux transporters, and thus, its use for the treatment of human diseases is limited (Chen et al., 2009).

During the formulation of baohuoside I-loaded mixed micelles, the prescription of preparation was investigated. For preparation of DTBM, TPGS mass fraction, a feeding of baohuoside I, and the amount of water were used as three variables at five levels for optimization in the design, taking two responses, including the encapsulation ratio (ER%) and the size of particles. The encapsulation efficiency of the micelles increased with an increase in the DDAB ratio in carrier, and the particle size increased significantly. Thus, the optimal formulation was the once in which the ratio of DDAB:TPGS:baohuoside I was 1:4:1 (w/w) and volume of water was 5 mL when the concentration of baohuoside I was 10 mg. DPBM had smaller particle size and higher ER%. Since HA can bind to by DDAB positive and negative charges, the ratio of HA and DDAB was examined by determining the response of particles size and zeta potential. The modification of HA can increase the particle size of the micelles. Therefore, the optimum ratio of HA: DDAB was 1:5 (w/w).

Particle size is important for developing nanoscale drug delivery systems targeting specific sites of the body. Small particles can avoid uptake by the RES and the mononuclear phagocyte system (MPS). Particles ranging from ∼10 to 200 nm can leave the vascular bed and accumulate inside the interstitial space of the tumor based on the EPR effect (Fang et al., 2011). In addition, particles with a diameter range of ∼10–30 nm can avoid the accelerated blood clearance phenomenon and accordingly achieve the long cycle effect (Koide et al., 2008,2012). These physicochemical properties endow the micellar vector with a potential beneficial behavior *in vivo*.

HA is one of the main components of the extracellular matrix and has physiological functions such as moisture retention in the skin and moisturizing effect (Witting et al., 2015). However, a previous study showed that high levels of HA are present in the margins of solid tumor tissue, and the HA receptors CD44 and RHAMM are highly expressed on the surface of the tumor cells. The HA signal transduction pathway is important for tumor growth, adhesion and metastasis. CD44 receptor-mediated endocytosis of HA induces invasion of the peripheral tissue of the tumor and distant metastasis. CD44 receptors are also expressed in normal tissues and primary cells, but the CD44 receptor is present in an inactive silent state in the primary cells of normal tissue, and the HA affinity of these cells is much lower than that of the tumor cells. The RHAMM and CD44 receptors enable delivery of chemotherapeutic drugs to solid tumors (Tran et al., 2014; Park et al., 2014,2016).

**Figure 5.**
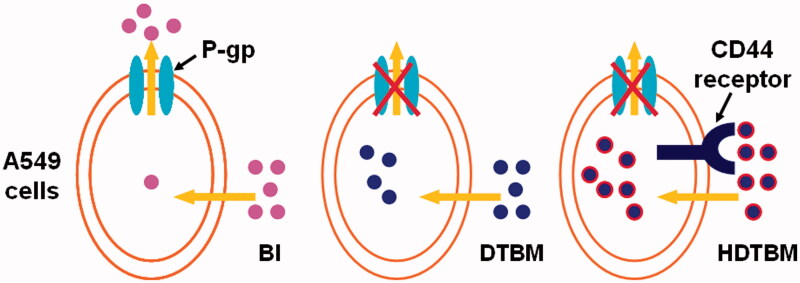
Graphical representation of internalization and accumulation of free drug and micelles modified by TPGS and HA in resistant A549 cells.

The IC50 value reflected the MDR reversing ability of certain formulations. Free baohuoside I was easily effluxed from cells via the P-gp. The IC50 values of other formulations were lower than that for free baohuoside I, which indicated that the mixed micelles had MDR reversing ability. Our results indicate that the superior cytotoxicity of HA/baohuoside I-M might be attributed to the drastic increase in drug accumulation in A549 cells both by reduced efflux and increased uptake (Figure 5). Free baohuoside I was taken up by the cells via passive diffusion, while the hydrophobically modified HA micelles increased uptake of the micelles via CD44-receptor mediated endocytosis, thus enhancing the inhibition of cell proliferation by baohuoside I. In addition, the mixed micelles without drug showed antiproliferative effects when the concentration of micellar materials was higher than 50 mg/mL, which might be explained by the antitumor effect of TPGS by activating mitochondria-specific apoptotic pathways in tumor cells.

A combined effect of the passive targeting by HA and the enhanced sensitization of MDR cancer by TPGS to baohuoside I could be the main reason for the significant suppression of tumor growth in the mixed micelle group. Because of the suitable size and the architecture of HA-modified mixed micelle, the micellar system could achieve targeted delivery and prolonged blood circulation effect, which led to an increase in the local concentration of baohuoside I in the tumor tissue via the EPR effect. Drastic sensitization of MDR tumors conferred by TPGS copolymers also enhanced the uptake of baohuoside I in tumor cells.

## Conclusion

In this study, we designed mixed micelles consisting of DDAB, TPGS and those modified with HA to entrap the poorly soluble anticancer drug baohuoside I. The baohuoside I mixed micelles (HDTBM) had 85% higher encapsulation efficiency and particle size around 30 nm. Compared to free baohuoside I, baohuoside I from HDTBM showed an initial burst release followed by controlled release and increased the cellular uptake and cytotoxicity *in vitro*. HDTBM showed markedly higher antitumor efficacy than baohuoside I in *in vivo* studies. In this study, we showed that HA/DDAB/TPGS mixed micelles significantly increased the antitumor and targeting efficacy, and they may be a potential delivery system to maximize the use of baohuoside I as a therapeutic agent for humans.
